# Fortieth Anniversary Virtual Issues: Microbial Diversity and the Tree of Life

**DOI:** 10.1093/molbev/msae069

**Published:** 2024-04-25

**Authors:** Casey McGrath

To celebrate the 40th anniversary of the first issue of *Molecular Biology and Evolution*, the Society for Molecular Biology and Evolution (SMBE) journals are launching a series of virtual issues in 2024. These virtual issues contain selected papers published in the society's journals, *Molecular Biology and Evolution* and *Genome Biology and Evolution*. Each virtual issue will be accompanied by a Perspective that highlights the historic and contemporary contributions of our journals to a specific topic in molecular evolution.

April's Perspective, titled “Microbial diversity and open questions about the deep Tree of Life,” appears in *Genome Biology and Evolution* and was written by Laura Eme and Daniel Tamarit. The accompanying virtual issues—one for *Molecular Biology and Evolution* and one for *Genome Biology and Evolution*—include noteworthy papers on microbial diversity and the Tree of Life published in the two journals and can be found at SMBE's 40th anniversary site.

